# Supplementary material for: In vivo bioluminescence imaging of natural bacteria within deep tissues via ATP-binding cassette sugar transporter
Source: Nat Commun. 2023 Apr 22;14:2331. doi: 10.1038/s41467-023-37827-9 (PMC10122673; doi:10.1038/s41467-023-37827-9)
Supplement: Supplementary file 1 — Supplementary Information [file 41467_2023_37827_MOESM1_ESM.pdf]

Supporting Information

In vivo bioluminescence imaging of natural bacteria within deep tissues *via* ATP-binding cassette sugar transporter

Qian Zhang,[1,‡] Bin Song,[1,‡] Yanan Xu,[1,‡] Yunmin Yang,[1] Jian Ji,[2] Wenjun Cao,[2] Jianping Lu,[1] Jiali Ding,[1] Haiting Cao,[1] Binbin Chu,[1] Jiaxu Hong,[2,*] Houyu Wang[1,*] & Yao He[1,*]

[1]Suzhou Key Laboratory of Nanotechnology and Biomedicine, Institute of Functional Nano & Soft Materials & Collaborative Innovation Center of Suzhou Nano Science and Technology (NANO-CIC), Soochow University, Suzhou 215123, China

[2]Department of Ophthalmology and Vision Science, Shanghai Eye, Ear, Nose and Throat Hospital, Fudan University, Shanghai, China.

*Corresponding authors. Email: jiaxu.hong@fdeent.org; houyuwang@suda.edu.cn; yaohe@suda.edu.cn

‡ These authors contributed equally.

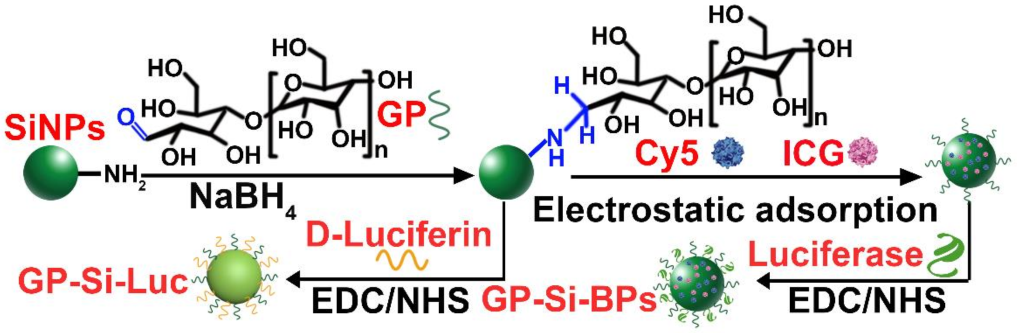

Supplementary Fig. 1. A scheme illustrating the synthesis of GP, Cy5, ICG and luciferase-modified silicon nanoparticles (SiNPs) (GP-Si-BPs) and the synthesis of GP, D-luciferin-modified SiNPs (GP-Si-Luc) step by step.

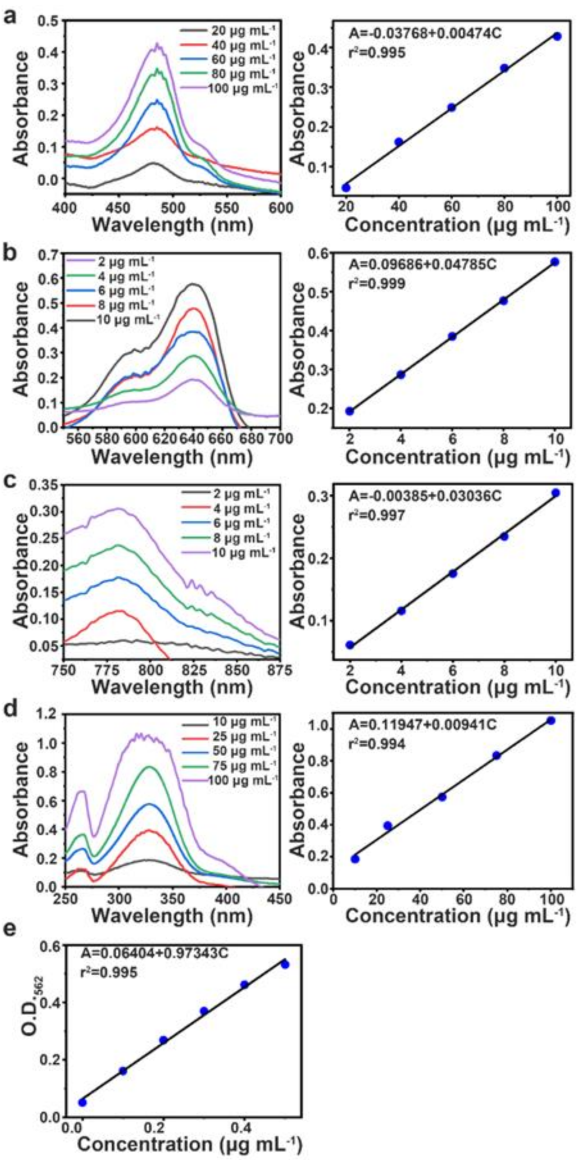

Supplementary Fig. 2. Quantification curves of GP, Cy5, ICG, D-Luciferin and Luciferase. a, UV-vis absorption spectra of GP with various concentrations ranged from 20 to 100 μg mL^{-1} and corresponding calibration curve. **b,** UV-vis absorption spectra of Cy5 with various concentrations ranged from 2 to 10 μg mL^{-1} and corresponding calibration curve. **c,** UV-vis absorption spectra of ICG with various concentrations ranged from 2 to 10 μg mL^{-1} and corresponding calibration curve. **d,** UV-vis absorption spectra of D-luciferin with various concentrations ranged from 10 to 100 μg mL^{-1} and corresponding calibration curve. **e,** Calibration curve of luciferase protein using the BCA assay. Error bars represent the standard deviation obtained from three independent measurements. Source data are provided as a Source data file.

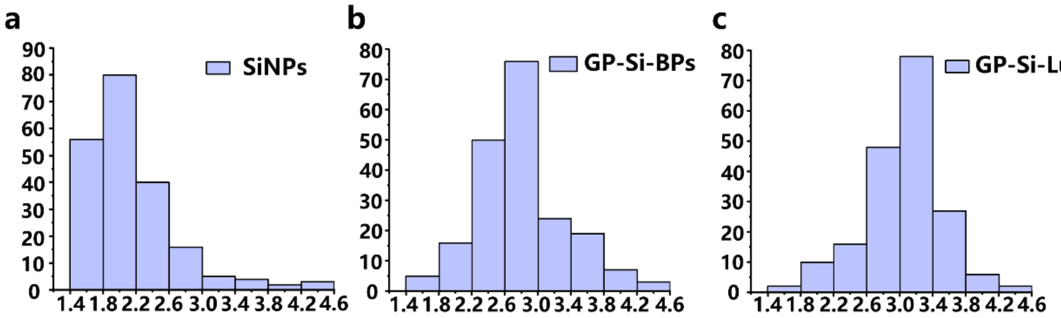

Supplementary Fig. 3. The size distribution of nanoparticles. **a,** Size distribution of SiNPs. **b,** Size distribution of GP-Si-BPs. **c,** Size distribution of GP-Si-Luc. The size distribution data were obtained by measuring 200 particles in corresponding TEM images (**Fig. 2a**). Accordingly, the average diameter of GP-Si-BPs was ~2.8 nm and the average diameter of GP-Si-Luc was ~ 2.9 nm, both of which was slightly larger than that of naked SiNPs (e.g., ~ 2.2 nm). Source data are provided as a Source data file.

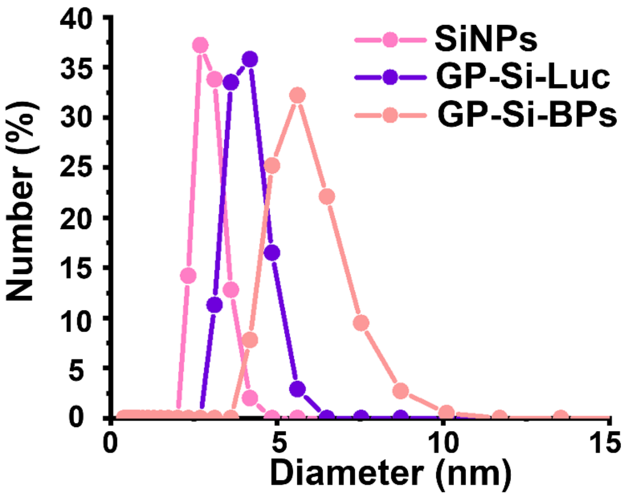

Supplementary Fig. 4. The DLS analysis of SiNPs, GP-Si-Luc and GP-Si-BPs. The DLS was performed using a DynaPro DLS, which was made by Malvern Corp, U.K. (ZEN3690). One mL of GP-Si-BPs, GP-Si-Luc or pure SiNPs sample was transferred into an exclusive vitreous for DLS measurements. Experiment parameters were as follows: scan times: 100; dispersant: water; temperature: 25°C; viscosity: 0.8872 cP; RI: 1.330; and dielectric constant: 78.5. Accordingly, the hydrodynamic diameter of GP-Si-BPs, GP-Si-Luc and pure SiNPs measured by DLS was ~ 5.6 nm, ~ 4.1 nm and ~ 3.1 nm. Source data are provided as a Source data file.

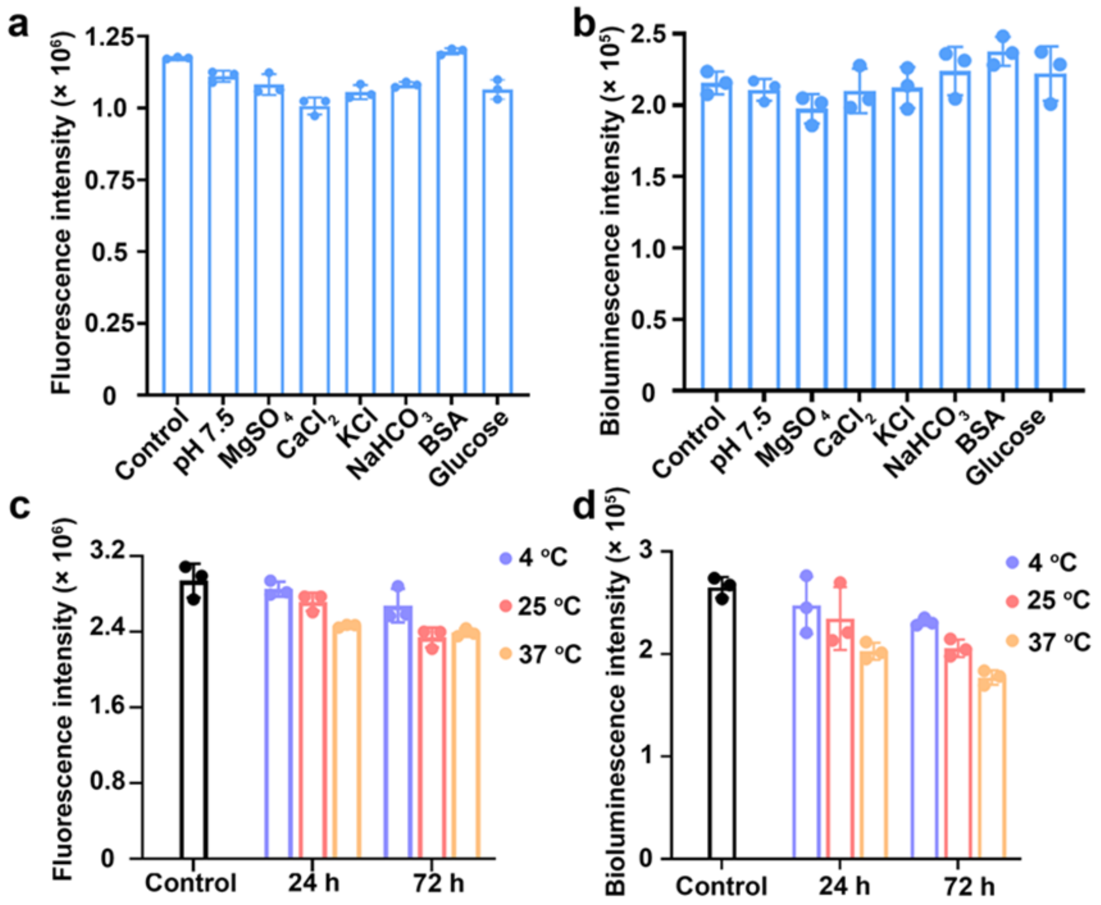

Supplementary Fig. 5. Stability test of GP-Si-BPs in nanoagents. a, The fluorescence intensity of ICG in GP-Si-BPs tested in various kinds of solutions with pH 7.5, as well as different kinds of solutions containing various intracellular species (e.g., 2 mM $MgSO_4$, 2 mM $CaCl_2$, 150 mM KCl, 10 mM $NaHCO_3$, 20 mM glucose and 1 mM bovine serum albumin (BSA)) (mean ± SD, n = 3). **b,** The bioluminescence intensity of GP-Si-BPs in nanoagents is tested in various kinds of solutions with pH 7.5, as well as different kinds of solutions containing various intracellular species (e.g., 2 mM $MgSO_4$, 2 mM $CaCl_2$, 150 mM KCl, 10 mM $NaHCO_3$, 20 mM glucose and 1 mM bovine serum albumin (BSA)) (mean ± SD, n = 3). **c,** The fluorescence intensity of GP-Si-BPs in PBS after storing at varied temperatures for 24 hours and for 72 hours (mean ± SD, n = 3). **d,** The bioluminescence intensity of GP-Si-BPs in PBS after storing at varied temperatures for 24 hours and for 72 hours (mean ± SD, n = 3). All error bars represent the standard deviation determined from three independent assays. Source data are provided as a Source Data file.

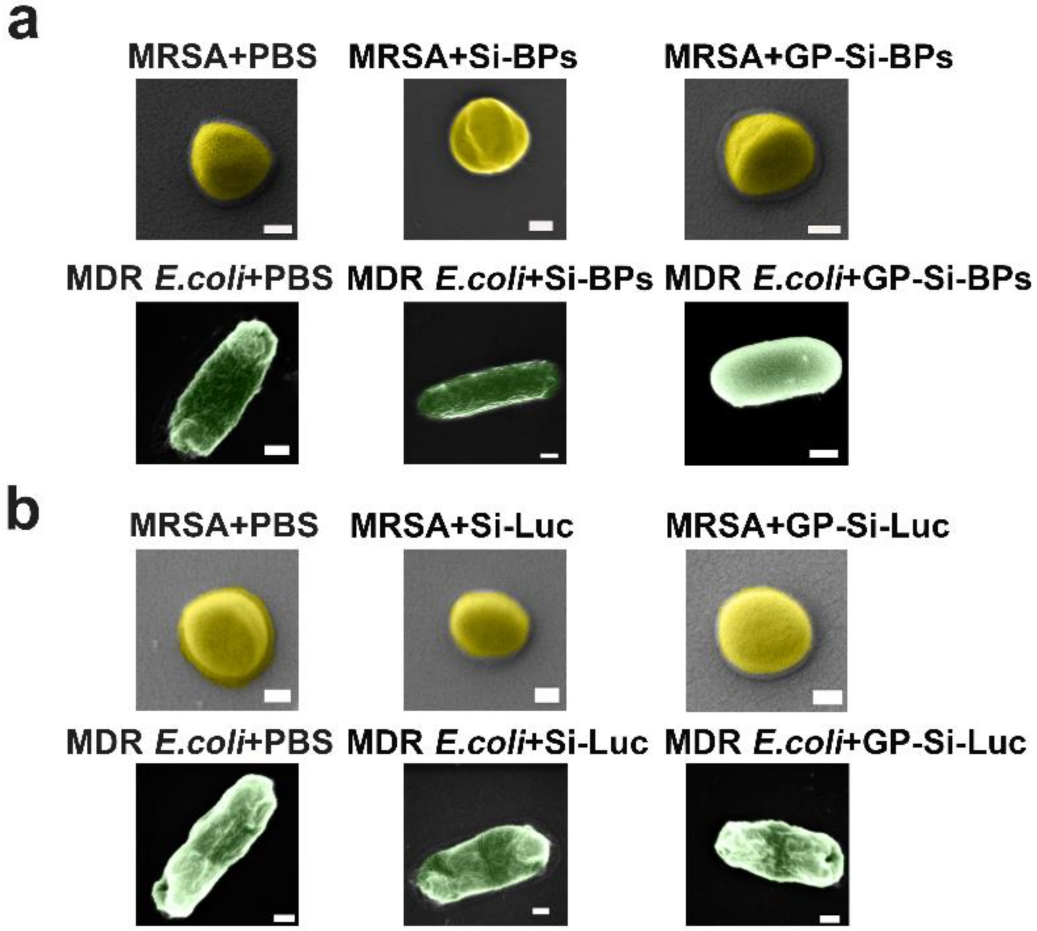

Supplementary Fig. 6. SEM images of bacteria treated with Trojan BLI probes. a, SEM images of MRSA or MDR *E. coli* treated by PBS (control), 0.06 mM of Si-BPs or GP-Si-BPs at 37 ºC for 2.5 h. After incubation, the treated bacteria were rinsed with PBS buffer for several times. Scale bar, 200 nm. All imaging experiments were repeated three times with similar results. **b,** SEM images of MRSA or MDR *E. coli* treated by PBS (control), 0.06 mM of Si-Luc or GP-Si-Luc at 37 ºC for 2.5 h. After incubation, the treated bacteria were rinsed with PBS buffer for several times. The bacterial cell concentration was ~1.0 $\times 10^7$ CFU. Scale bar, 200 nm. All imaging experiments were repeated three times with similar results.

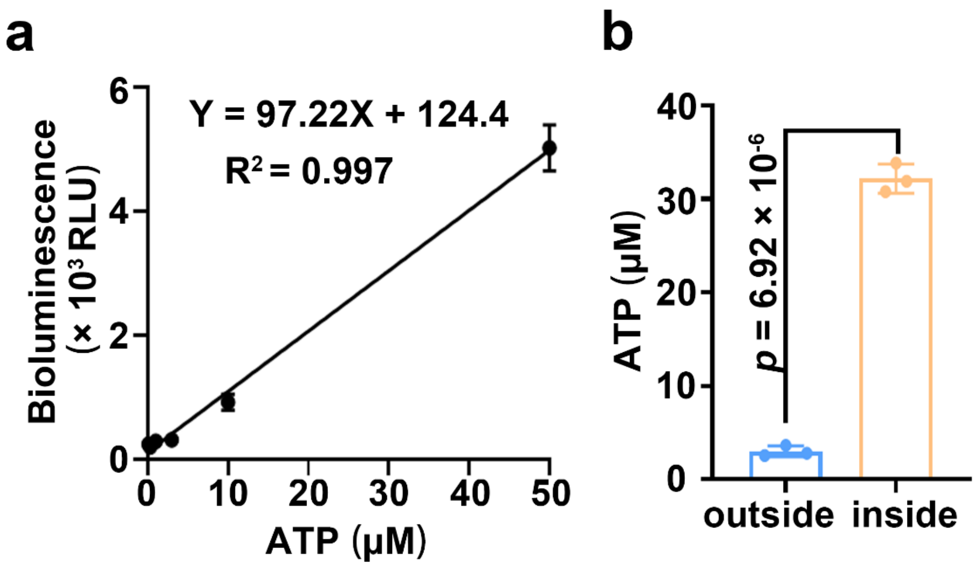

Supplementary Fig. 7. Comparison of ATP contents inside bacteria and outside bacteria determined by ATP assay kit. a, Linear correlation between luminescence intensities and ATP concentrations in ATP kit assay. **b**, Comparison of ATP contents inside MDR *E. coli* and outside MDR *E. coli*. After centrifuging the bacterial solution, the ATP kit was used to measure the bioluminescence intensity of the supernatant and the bacterial solution after equal volume PBS re-selection, and then inserted the detected bioluminescence intensity into the linear regression equation in **Supplementary Fig. 7a** to calculate the ATP content. The bacterial cell concentration was ~1.0 $\times 10^7$ CFU. Statistical analysis was performed using a one-way ANOVA analysis (mean ± SD, n = 3). Error bars represent the standard deviation obtained from three independent measurements. Source data are provided as a Source data file.

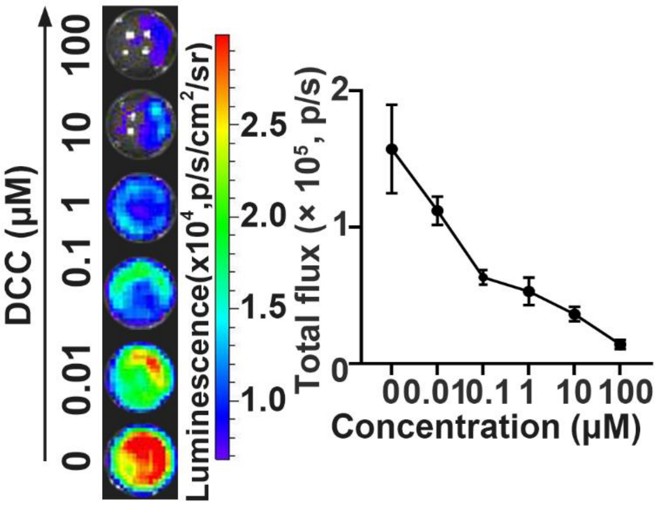

Supplementary Fig. 8. Attenuation of the luminescence at 0.06 mM GP-Si-BPs in the presence of 0.06 mM GP-Si-Luc by addition of the ATP inhibitor of dicyclohexylcarbodiimide (DCC). All imaging experiments were repeated three times with similar results (mean ± SD, n = 3). Error bars represent the standard deviation obtained from three independent measurements. Source data are provided as a Source data file.

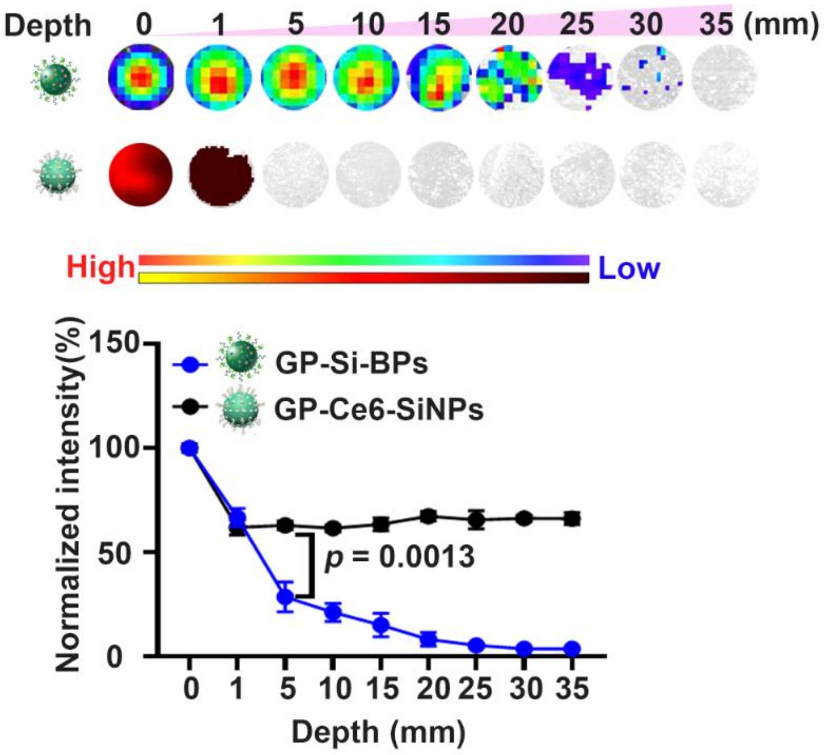

Supplementary Fig. 9. Comparison of tissue penetration depth between GP-Si-BPs and GP-Ce6-SiNPs. All imaging experiments were repeated three times with similar results. Statistical analysis was performed using a one-way ANOVA analysis (mean ± SD, n = 3). Error bars represent the standard deviation obtained from three independent measurements. Source data are provided as a Source data file.

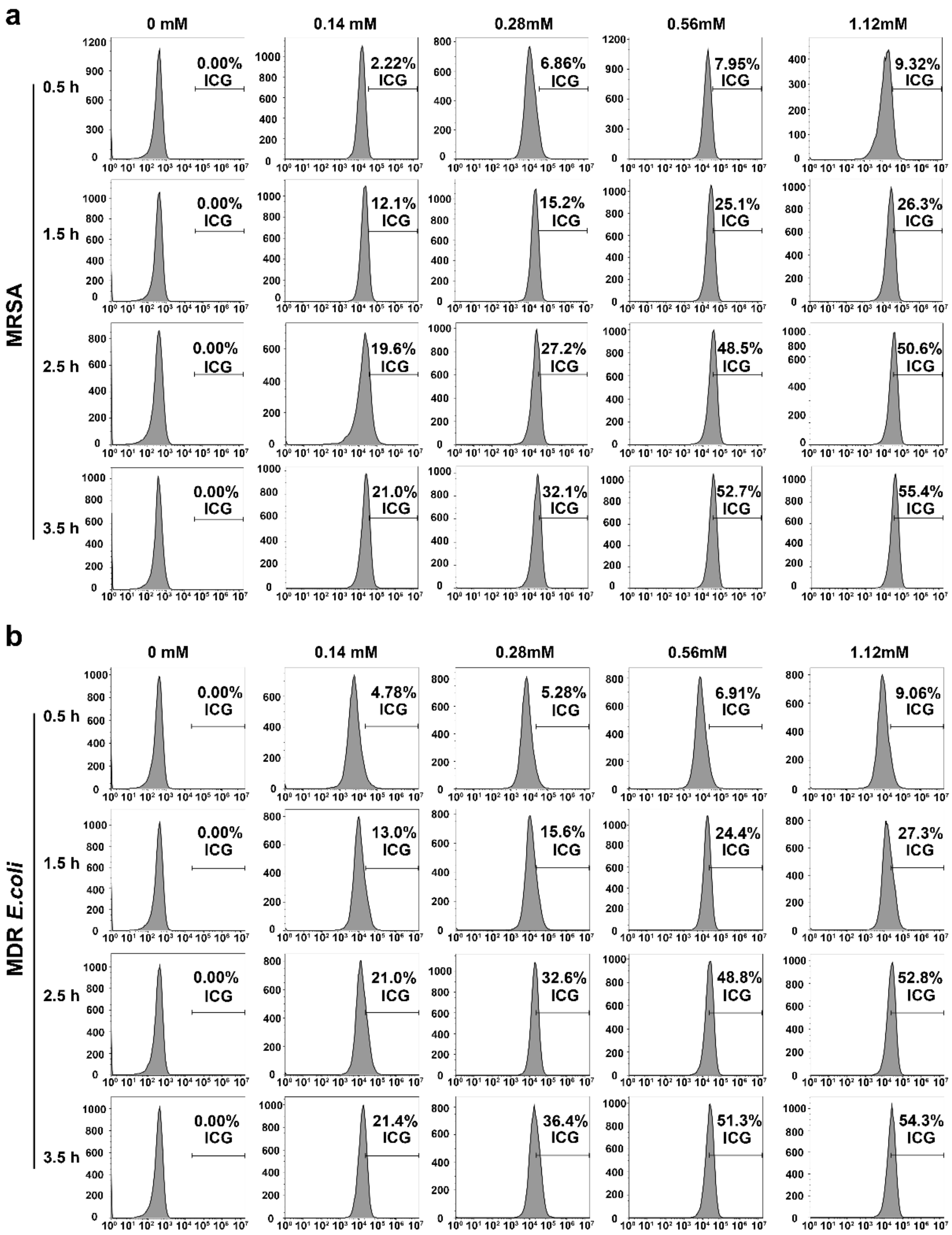

Supplementary Fig. 10. The effects of GP concentration and incubation time on the uptake of GP-Si-BPs by bacteria. a, Flow cytometry analysis of MRSA co-incubated with GP-Si-BPs with GP concentration (0, 0.14, 0.28, 0.56, 1.12 mM) at 37 °C for 0.5 h, 1.5 h, 2.5 h and 3.5 h, respectively. **b,** Flow cytometry analysis of MDR *E. coli* co-incubated with GP-GP-Si-BPs with GP concentration (0, 0.14, 0.28, 0.56, 1.12 mM) at 37 °C for 0.5 h, 1.5 h, 2.5 h and 3.5 h, respectively.

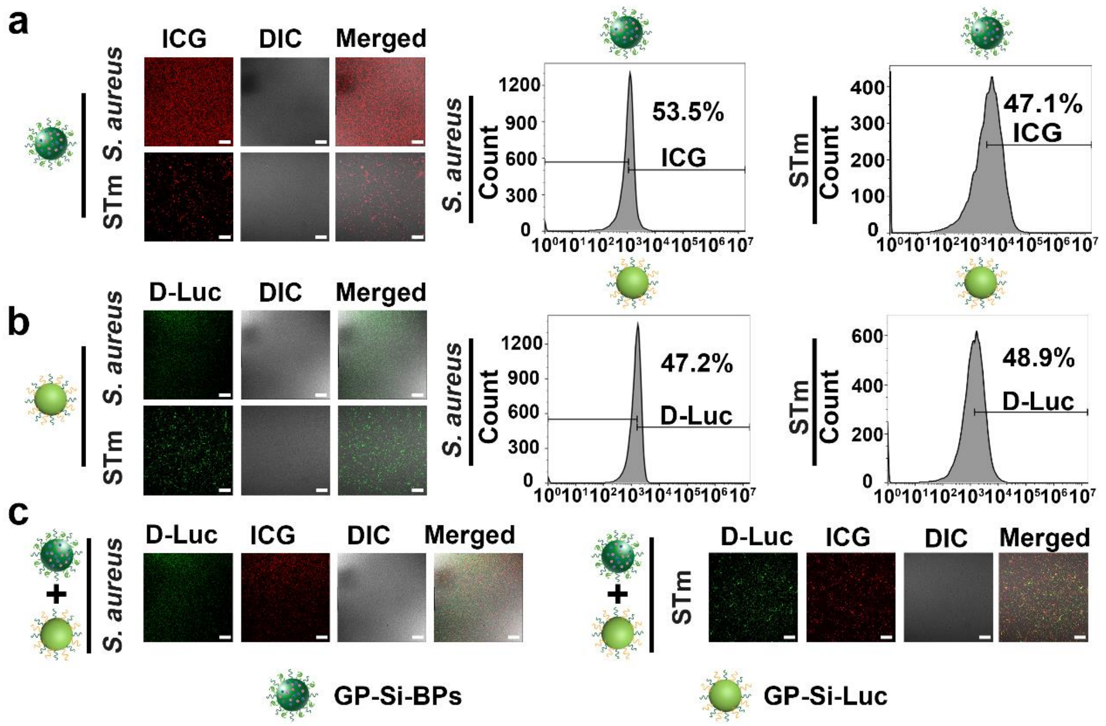

Supplementary Fig. 11. CLSM images of bacteria treated with GP-Si-BPs, GP-Si-Luc or GP-Si-BPs + GP-Si-Luc. a, Confocal images of *S. aureus* or *Salmonella typhimurium* (STm) after the incubation with 0.06 mM GP-Si-BPs and corresponding flow cytometry analysis of uptake rates. **b,** Confocal images of *S. aureus* or STm after the incubation with 0.06 mM GP-Si-Luc and corresponding flow cytometry analysis of uptake rates. **c,** Confocal images of *S. aureus* or STm after the incubation with 0.06 mM GP-Si-BPs + GP-Si-Luc. After incubation, the treated bacteria were rinsed with PBS buffer for several times. The bacterial cell concentration is ~10^7 CFU. Scale bar, 25 μm. All imaging experiments were repeated three times with similar results.

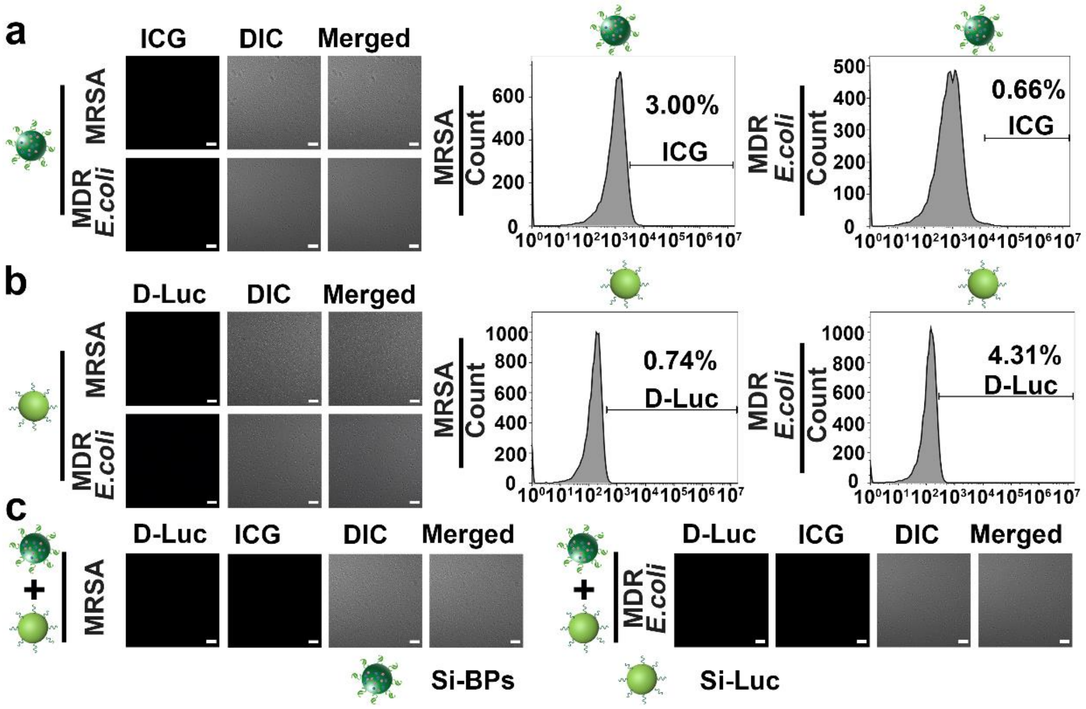

Supplementary Fig. 12. CLSM images of bacteria treated with Si-BPs, Si-Luc or Si-BPs + Si-Luc. a, Confocal images of MRSA or MDR *E. coli* after the incubation with 0.06 mM Si-BPs and corresponding flow cytometry analysis of uptake rates. **b,** Confocal images of MRSA or MDR *E. coli* after the incubation with 0.06 mM Si-Luc and corresponding flow cytometry analysis of uptake rates. **c,** Confocal images of MRSA or MDR *E. coli* after the incubation with 0.06 mM Si-BPs + Si-Luc. After incubation, the treated bacteria were rinsed with PBS buffer for several times. The bacterial cell concentration is ~10^7 CFU. Scale bar, 25 μm. All imaging experiments were repeated three times with similar results.

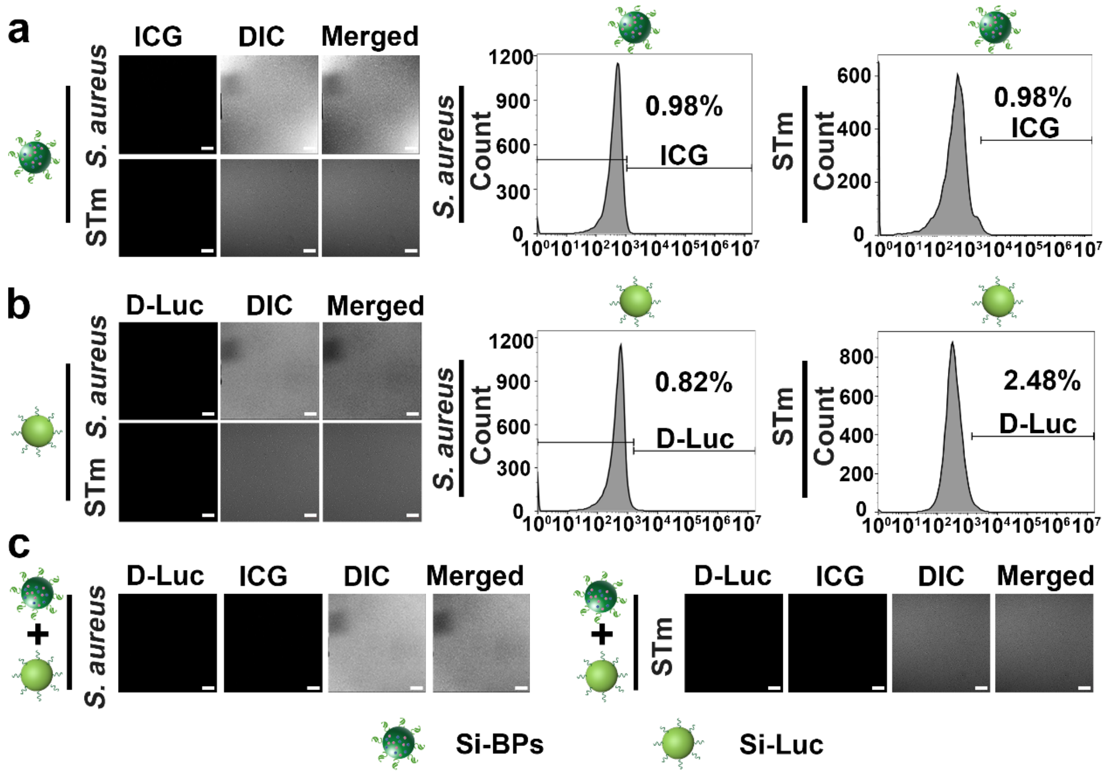

Supplementary Fig. 13. CLSM images of bacteria treated with Si-BPs, Si-Luc and Si-BPs + Si-Luc. a, Confocal images of *S. aureus* or *Salmonella typhimurium* (STm) after the incubation with 0.06 mM Si-BPs and corresponding flow cytometry analysis of uptake rates. **b,** Confocal images of *S. aureus* or STm after the incubation with 0.06 mM Si-Luc and corresponding flow cytometry analysis of uptake rates. **c,** Confocal images of *S. aureus* or STm after the incubation with 0.06 mM Si-BPs + Si-Luc. After incubation, the treated bacteria were rinsed with PBS buffer for several times. The bacterial cell concentration is ~10^7 CFU. Scale bar, 25 μm. All imaging experiments were repeated three times with similar results.

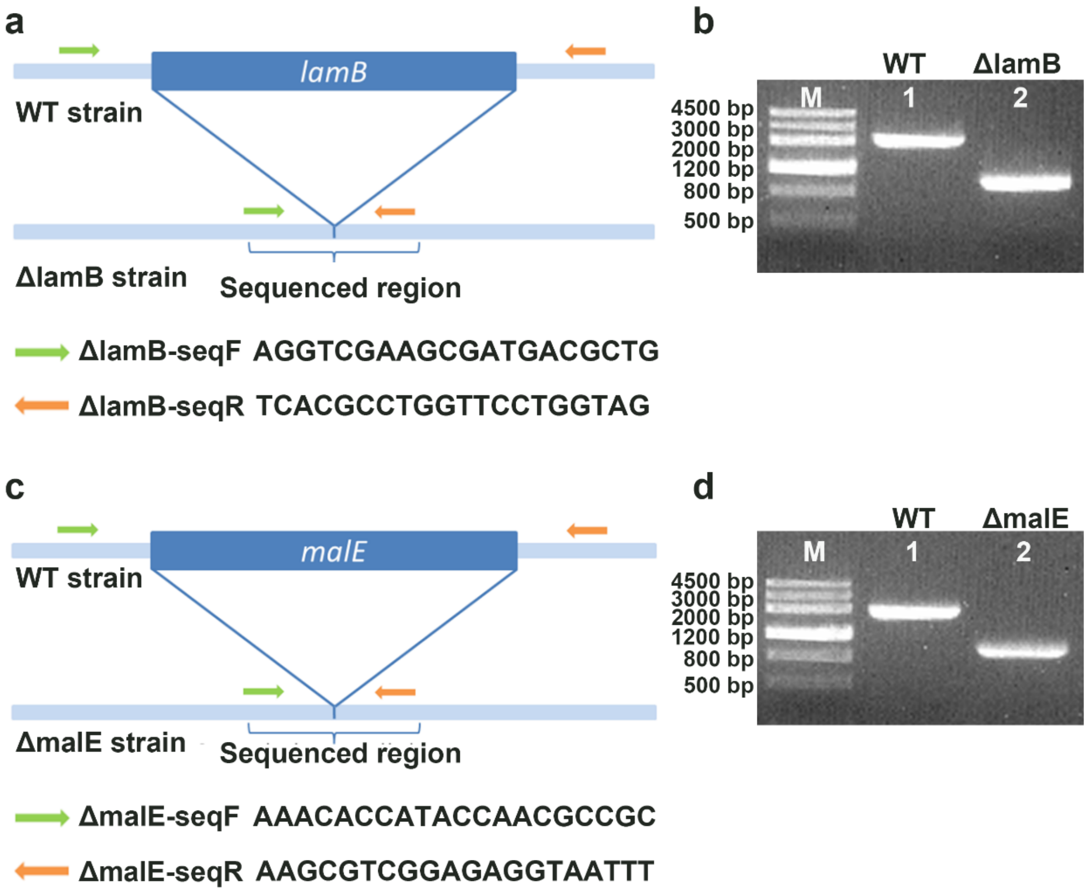

Supplementary Fig. 14. Confirmation of the successful construction of ΔlamB and ΔmalE. a, A scheme illustrating the location of primers of ΔlamB-seqF and ΔlamB-seqR relative to lamB. **b**, Confirmation of lamB knockout by PCR. All imaging experiments were repeated three times with similar results. **c**, A scheme illustrating the location of primers of ΔmalE-seqF and ΔmalE-seqR relative to malE. **d**, Confirmation of malE knockout by PCR. All imaging experiments were repeated three times with similar results.

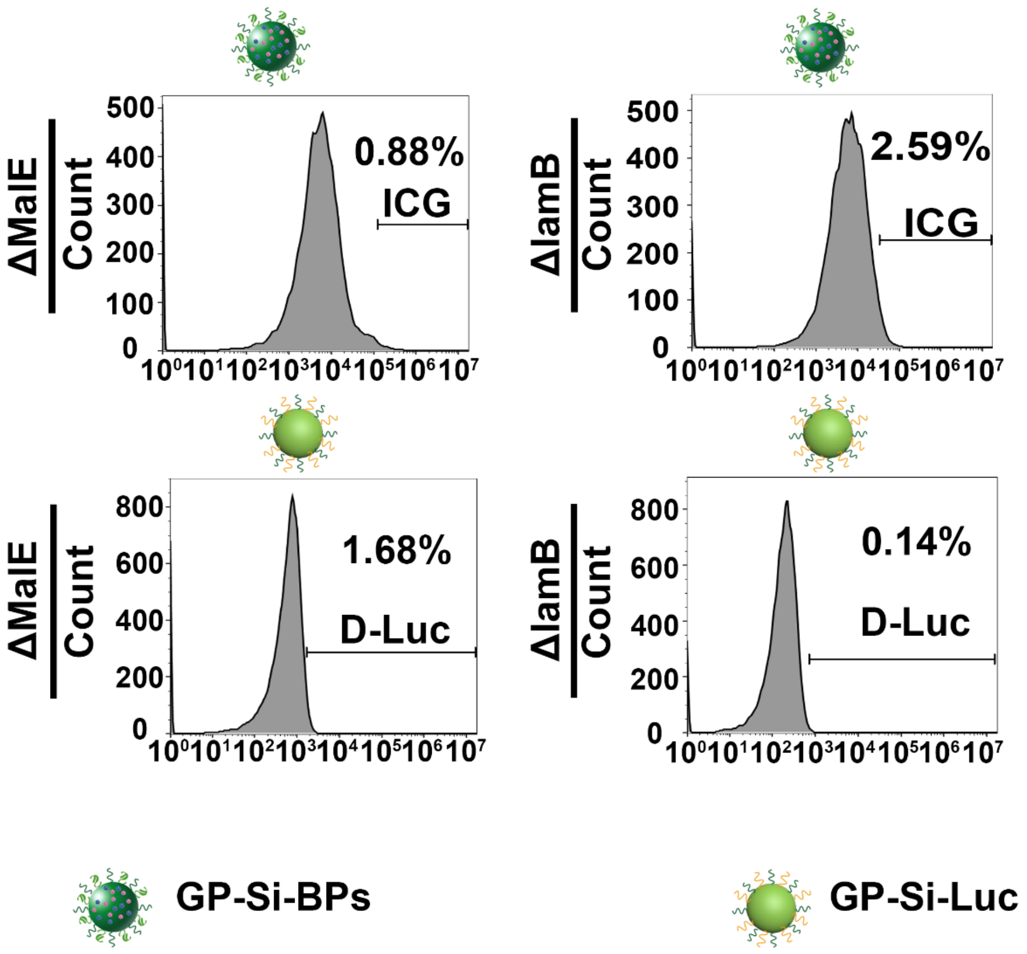

Supplementary Fig. 15. Flow cytometry analysis of uptake rates of bacteria mutants of ΔlamB and ΔmalE after incubation with 0.06 mM GP-Si-BPs or GP-Si-Luc. The bacterial cell concentration is ~10^7 CFU.

-

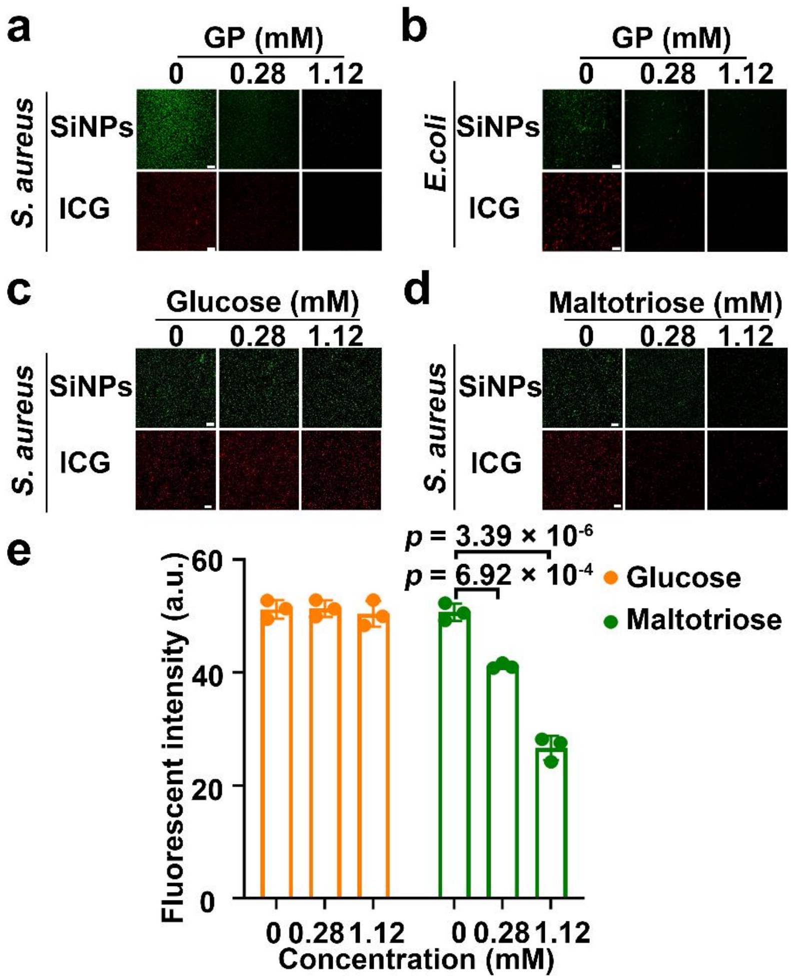

Supplementary Fig. 16. CLSM images of the GP-Si-BPs-treated bacteria after treatments of pure GP, glucose and maltotriose with different concentrations. **a-b,** Confocal fluorescence images of *S. aureus* (**a**) or *E. coli* (**b**) incubated with GP with different concentrations (e.g., 0, 0.28, 1.12 mM) for 5 min and then incubated with 0.06 mM GP-Si-BPs for another 2.5 h. **c-d,** Confocal fluorescence images of *S. aureus* incubated with glucose (**c**) or maltotriose (**d**) with different concentrations (e.g., 0, 0.28, 1.12 mM) for 5 min and then incubated with 0.06 mM GP-Si-BPs for another 2.5 h. **e,** Corresponding histograms of fluorescent intensity images of *S. aureus* incubated with glucose or maltotriose. The bacterial cell concentration is ~10^7 CFU. Scale bar, 25 μm (mean ± SD, n = 3). Statistical analysis was performed using a one-way ANOVA analysis. Error bars represent the standard deviation obtained from three independent measurements. All imaging experiments were repeated three times with similar results. Source data are provided as a Source data file.

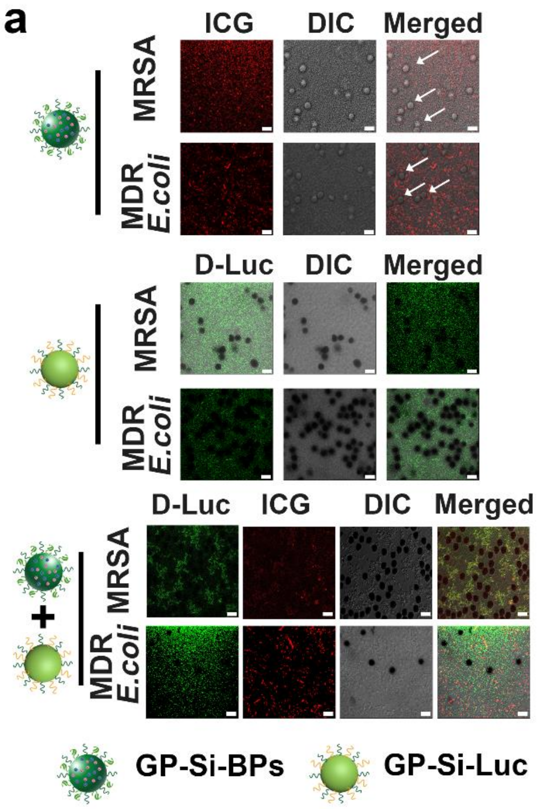

Supplementary Fig. 17. Evaluation of the selectivity of GP-Si-BPs and GP-Si-Luc for bacteria over mammalian cells. a, Confocal fluorescence images of pure human blood, the mixture of human blood and MRSA or MDR *E. coli* after incubation with GP-Si-BPs, GP-Si-Luc and GP-Si-BPs + GP-Si-Luc. Arrows indicate red blood cells (RBCs). Scale bar, 25 µm. All imaging experiments were repeated three times with similar results.

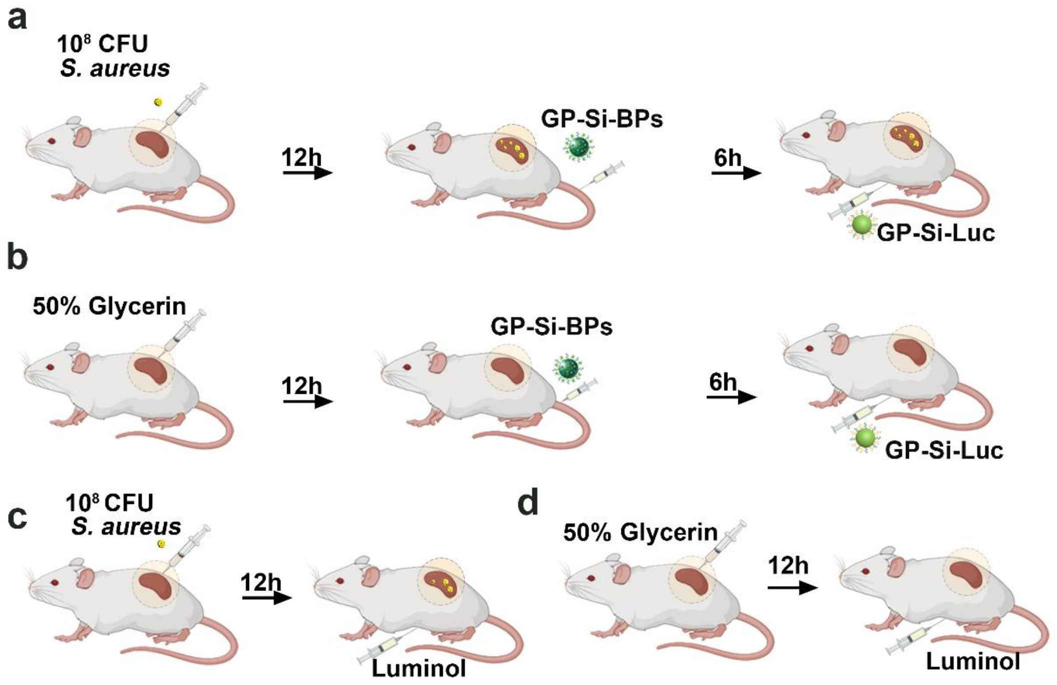

Supplementary Fig. 18. schemes illustrating *in vivo* imaging in mouse models of nephritis based on the proposed Trojan horse strategy. a, A scheme illustrating the bioluminescent imaging of *S. aureus*-induced nephritis in mice by using the Trojan BLI probes. The treated mice (Female, 6-8 weeks old，n = 3) were intravenously injected with 200 μL of 0.06 mM GP-Si-BPs or Si-BPs at 12-h post-injection of 25 μL of *S. aureus*. After 6 hours, these mice were intraperitoneally injected with GP-Si-Luc or Si-Luc (0.06 mM, 200 μL), followed by *in vivo* imaging by using an optical imaging system (IVIS Lumina III) at 15-min post-injection. The actual *S. aureus* concentration at the infection site during imaging was ~1.0×10^8 CFU, which was determined through kidney tissue harvesting, homogenization, and culturing with CFU count. **b,** A scheme illustrating the bioluminescent imaging of 50% glycerin-induced nephritis in mice by using the Trojan BLI probes. The mice (Female, 6-8 weeks old, n = 3) were *in situ* injected with 25 μL 50% (v/v) glycerin, followed by intravenous injection of 200 μL of 0.06 mM GP-Si-BPs at 12-h post-injection. Six hours later, GP-Si-Luc (0.06 mM) were intraperitoneally injected into these mice, and the infected sites were imaged by an *in vivo* optical imaging system (IVIS Lumina III) at 15-min post-injection. **c,** A scheme illustrating the luminescent imaging of *S. aureus*-induced nephritis in mice by using luminol. **d,** A scheme illustrating the luminescent imaging of 50% glycerin-induced

nephritis in mice by using luminol. The 200 μL of 282 mM luminol were intraperitoneally injected into *S. aureus* nephritis-bearing mice or glycerin nephritis-bearing mice, followed by imaging by using an *in vivo* optical imaging system (IVIS Lumina III) at 10-min post-injection.

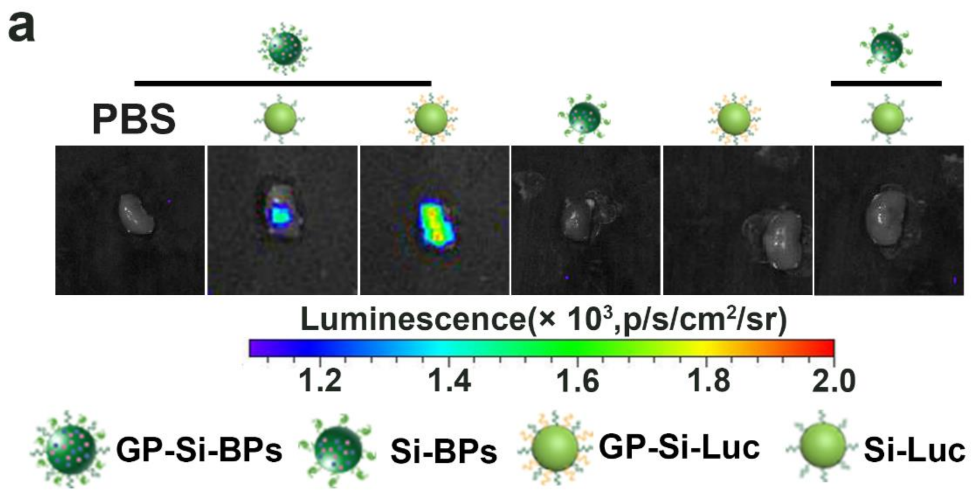

Supplementary Fig. 19. *Ex vivo* bioluminescence imaging of kidney tissues isolated from *S. aureus* (~1.0 × 10^8 CFU) nephritis-bearing mice with different treatments as indicated. All imaging experiments were repeated three times with similar results.

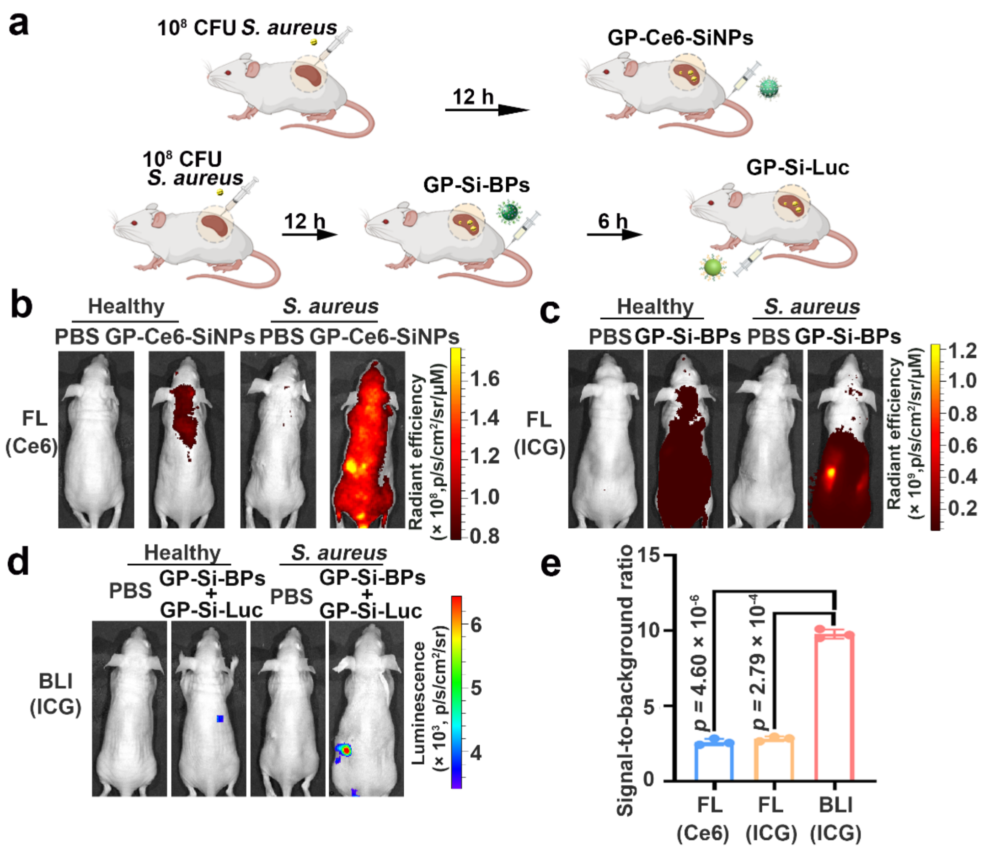

Supplementary Fig. 20. Comparison of GP-Ce6-SiNPs system (simply excitation of Ce6), Trojan BLI system (GP-Si-BPs + GP-Si-Luc), and ICG-based NIR emission imaging (simply excitation of ICG in GP-Si-BPs) in the imaging of *S. aureus*-induced nephritis. **a**, A scheme illustrating the optical imaging of *S. aureus*-induced nephritis in mice by using GP-Ce6-SiNPs system, Trojan BLI system or ICG-based NIR emission imaging. **b**, Fluorescence imaging of healthy mice and *S. aureus* nephritis-bearing mice treated with GP-Ce6-SiNPs or not (excitation: 460 nm, emission: 670 nm). **c**, Fluorescence imaging of healthy mice and *S. aureus* nephritis-bearing mice treated with GP-Si-BPs or not (excitation: 780 nm, emission: 845 nm). **d**, Bioluminescence imaging of healthy mice and *S. aureus* nephritis-bearing mice treated with GP-Si-BPs + GP-Si-Luc or not (emission: 845 nm). **e**, Corresponding signal-to-noise ratios obtained by GP-Ce6-SiNPs system, ICG-based NIR emission imaging and Trojan BLI system in the imaging of *S. aureus*-induced nephritis in mice (mean ± SD, n = 3). Statistical analysis was performed using a one-way ANOVA analysis. Error bars represent the standard deviation obtained from three independent measurements. Source data are provided as a Source data file.

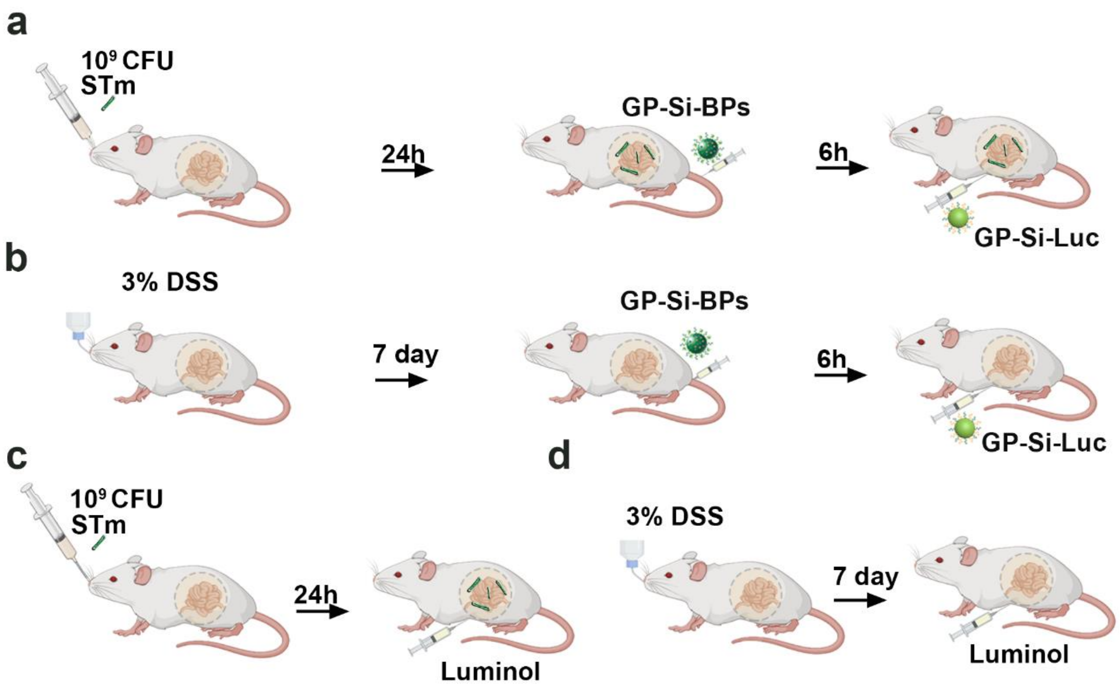

Supplementary Fig. 21. Schemes illustrating *in vivo* imaging in mouse models of colitis based on the proposed Trojan horse strategy. a, A scheme illustrating the bioluminescent imaging of STm-induced colitis in mice based on the Trojan BLI probes. The 100 μL STm were oral administrated into the mice (Female, 6-8 weeks old, n = 3), and 24 hours later, the treated mice were intravenously injected with 200 μL of 0.06 mM GP-Si-BPs or Si-BPs. At 6-h post-injection, the treated mice were intraperitoneally injected with GP-Si-Luc or Si-Luc (0.06 mM), followed by BLI by using an *in vivo* optical imaging system (IVIS Lumina III) at 15-min post-injection of substrates. The actual amount of STm at the infection site during imaging was determined as ~1.0 × 10^9 CFU, which was obtained through intestinal tissue harvesting, homogenization, and culturing with CFU count. **b,** A scheme illustrating the bioluminescent imaging of DSS-induced colitis in mice based on the Trojan BLI probes. **c,** A scheme illustrating the luminescent imaging of STm-induced colitis in mice based on luminol. **d,** A scheme illustrating the luminescent imaging of DSS-induced colitis in mice based on luminol. The 200 μL of 282 mM luminol were intraperitoneally injected into STm colitis-bearing mice or DSS colitis-bearing mice, followed by imaging by using an *in vivo* optical imaging system (IVIS Lumina III) at 10-min post-injection.

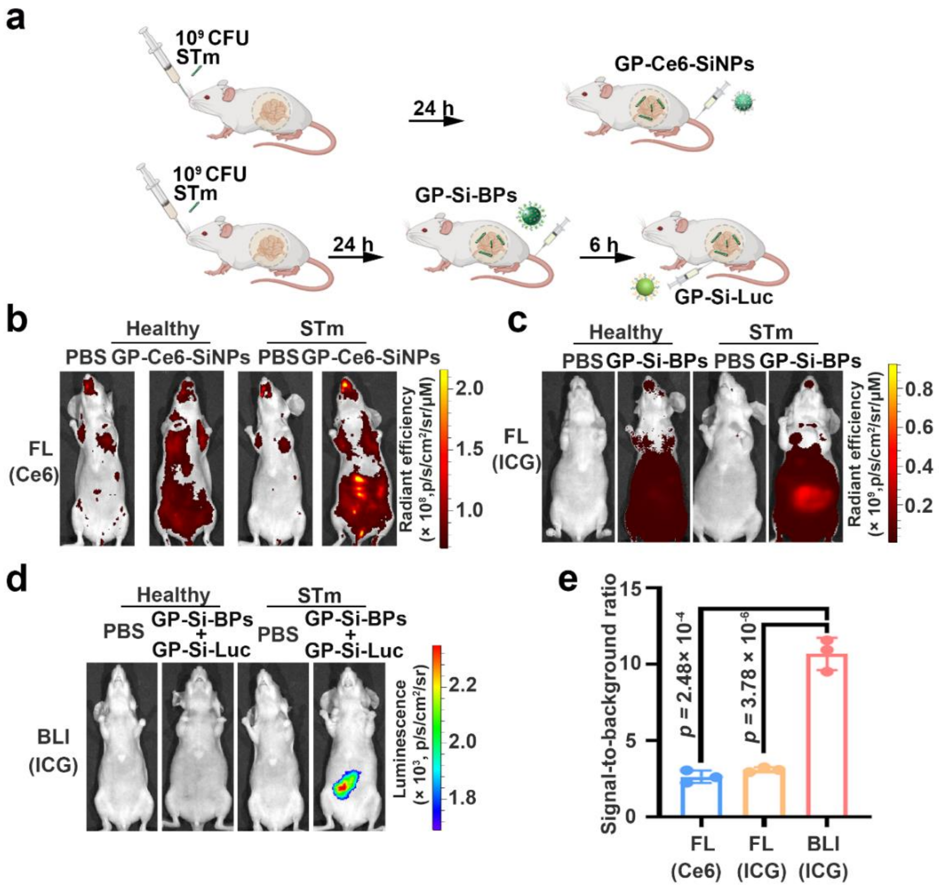

Supplementary Fig. 22. Comparison of GP-Ce6-SiNPs system (simply excitation of Ce6), Trojan BLI system (GP-Si-BPs + GP-Si-Luc), and ICG-based NIR emission imaging (simply excitation of ICG in GP-Si-BPs) in the imaging of STm-induced colitis. **a,** A scheme illustrating the luminescent imaging of STm-induced colitis in mice by using GP-Ce6-SiNPs system, Trojan BLI system or ICG-based NIR emission imaging. **b**, Fluorescence imaging of healthy mice and STm colitis-bearing mice treated with GP-Ce6-SiNPs or not (excitation: 460 nm, emission: 670 nm). **c**, Fluorescence imaging of healthy mice and STm colitis-bearing mice treated with GP-Si-BPs or not (excitation: 780 nm, emission: 845 nm). **d**, Bioluminescence imaging of healthy mice and STm colitis-bearing mice treated with GP-Si-BPs + GP-Si-Luc or not (emission: 845 nm). **e**, Corresponding signal-to-noise ratios obtained by GP-Ce6-SiNPs system, ICG-based NIR emission imaging and Trojan BLI system in the imaging of STm-induced colitis in mice (mean ± SD, n = 3). Statistical analysis was performed using a one-way ANOVA analysis. Error bars represent the standard deviation obtained from three independent measurements. Source data are provided as a Source data file.

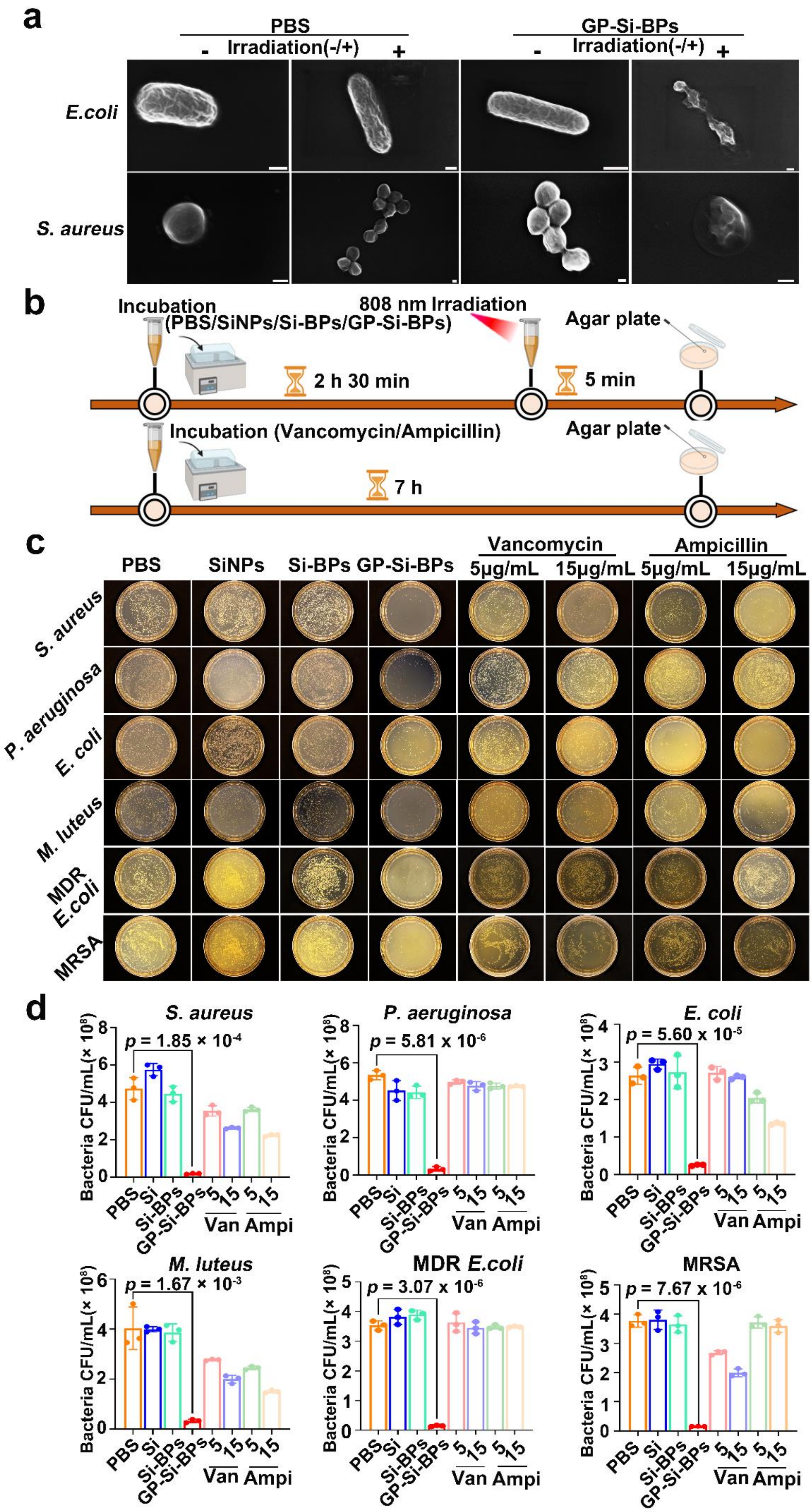
a
PBS
GP-Si-BPs
Irradiation(-/+)
E.coli
S. aureus
b
Incubation (PBS/SiNPs/Si-BPs/GP-Si-BPs)
808 nm Irradiation
Agar plate
2 h 30 min
5 min
Incubation (Vancomycin/Ampicillin)
7 h
c
PBS
SiNPs
Si-BPs
GP-Si-BPs
Vancomycin
Ampicillin
5μg/mL
15μg/mL
S. aureus
P. aeruginosa
E. coli
M. luteus
MDR E.coli
MRSA
d
S. aureus
p = 1.85 × 10-4
P. aeruginosa
p = 5.81 x 10-6
E. coli
p = 5.60 x 10-5
M. luteus
p = 1.67 × 10-3
MDR E.coli
p = 3.07 x 10-6
MRSA
p = 7.67 x 10-6
Bacteria CFU/mL(× 10⁸)
PBS Si Si-BPs GP-Si-BPs 5 15 5 15
Van Ampi

Supplementary Fig. 23. *In vitro* antibacterial activity based on the Trojan BLI probes. a, SEM images of *E. coli* or *S. aureus* treated with PBS or GP-Si-BPs with or without irradiation. Scale bar, 200 nm. **b**, A scheme illustrating the antibacterial evaluation of the developed strategy. **c**, Photographs of agar plates of *S. aureus*, *P. aeruginosa*, *E. coli*, *M. luteus*, multidrug-resistant *Escherichia coli* (MDR *E. coli*) and multidrug-resistant *Staphylococcus aureus* (MRSA) treated by PBS, SiNPs, Si-BPs, GP-Si-BPs (with irradiation of 808-nm laser, 1.0 W cm^{-2}, 5 min), vancomycin or ampicillin (with various concentration) and **d**, corresponding histograms of bacterial amounts (mean ± SD, n = 3). All imaging experiments were repeated three times with similar results. Statistical analysis was performed using a one-way ANOVA analysis. Source data are provided as a Source data file.

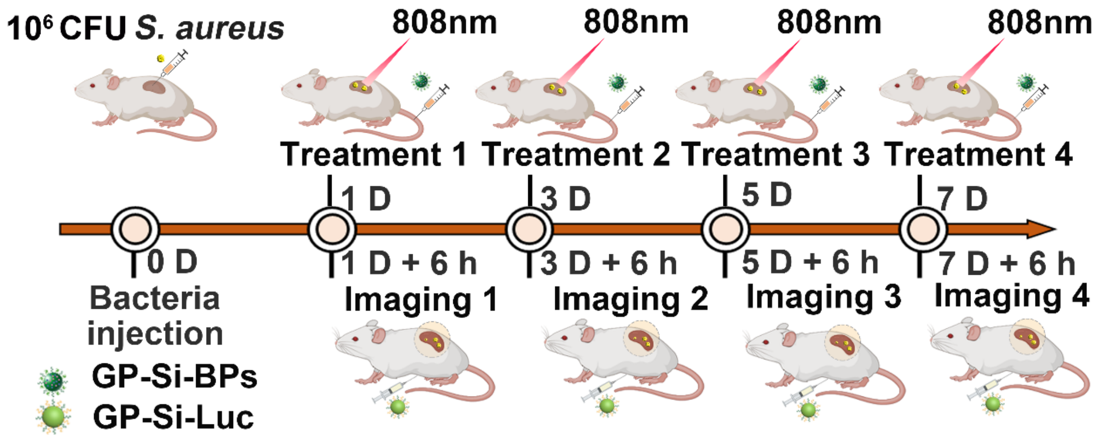

Supplementary Fig. 24. A scheme illustrating Trojan BLI strategy for therapy and imaging of bacterial nephritis-bearing mice. At 6-h post-injection of 1.0 $\times 10^6$ CFU of *S. aureus*, the infected mice (Female, 6-8 weeks old， n = 3) were intravenously injected with 200 μL of 0.06 mM GP-Si-BPs or PBS buffer on day 1 (Treatment 1), day 3 (Treatment 2), day 5 (Treatment 3) and day 7 (Treatment 4) respectively, and photothermal treatment (PTT) was performed under 808-nm laser irradiation 6 hours after each drug injection. And the photothermal treatment lasted for 5 minutes. Afterwards, mice were intraperitoneally injected with GP-Si-Luc after each irradiation for imaging to assay the therapeutic effect *in vivo*.

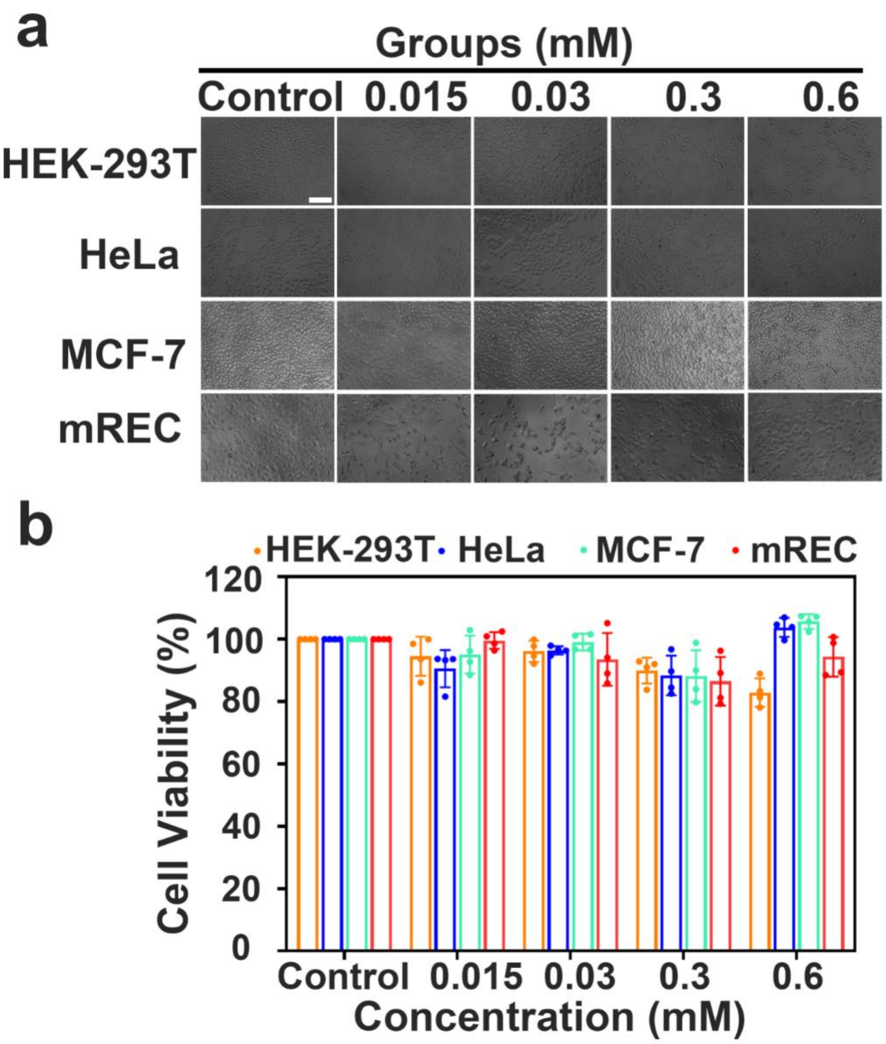

Supplementary Fig. 25. Evaluation of cytotoxicity of Trojan BLI probes. a, Morphology of HEK-293T, HeLa, MCF-7 and mREC cells treated with GP-Si-BPs + GP-Si-Luc (0.06 mM) for 24 h, respectively. **b**, Cell viability of HEK-293T, HeLa, MCF-7 and mREC cells treated with GP-Si-BPs + GP-Si-Luc with different dosages for 24 h, respectively (mean ± SD, n = 4). Scale bar, 50 μm. All imaging experiments were repeated four times with similar results. Source data are provided as a Source data file.

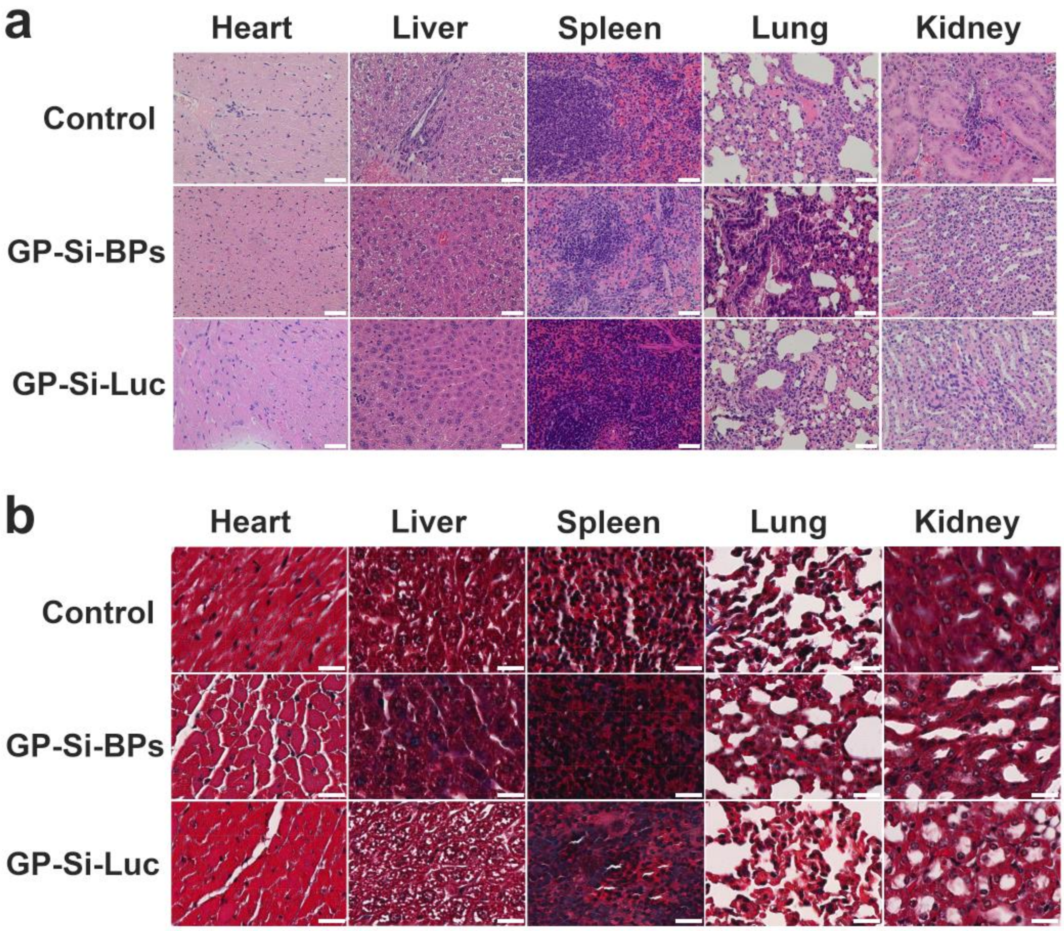

Supplementary Fig. 26. Representative histological images of major organs of healthy mice treated with 0.06 mM GP-Si-BPs or GP-Si-Luc for 24 h. **a**, H&E staining of different organs (heart, liver, spleen, lung and kidney) harvested from treated mice. Scale bar, 20 μm. All imaging experiments were repeated three times with similar results. **b**, Masson's trichrome staining of different organs (heart, liver, spleen, lung and kidney) harvested from treated mice. Scale bar, 20 μm. All imaging experiments were repeated three times with similar results.

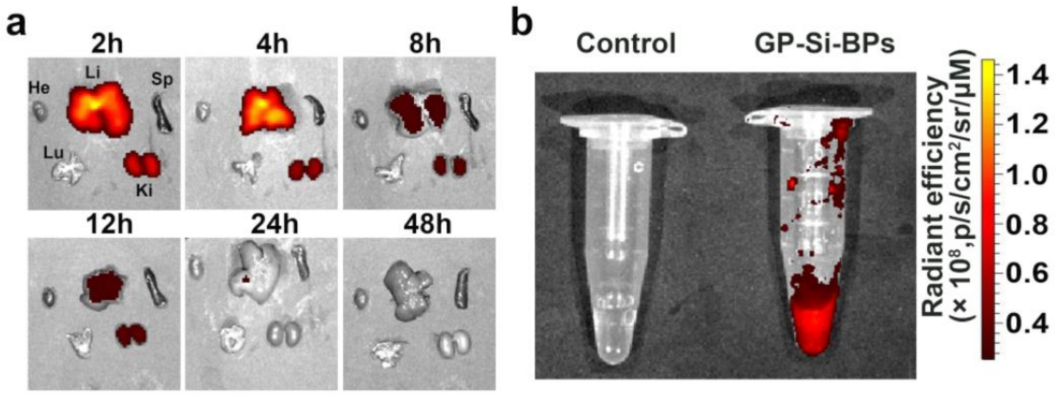

Supplementary Fig. 27. a, *Ex vivo* fluorescence imaging of organs resected from healthy mice after intravenous injection of 0.06 mM GP-Si-BPs at different time points. First row (from left to right): heart (He), liver (Li), spleen (Sp); second row (from left to right): lung (Lu), and kidney (Ki). **b,** *Ex vivo* fluorescence imaging of urine from healthy mice treated with PBS (control) or 0.06 mM GP-Si-BPs after 24 h post-injection.

Supplementary Table 1. Comparison of the features of glucose polymer (GP) modified imaging or therapeutic nanoagents.

Refs.	Features		
	Agents composition	Mechanisms of entry into bacteria	Functions
Nat. Commun. **10**, 4057 (2019)	GP and chlorin e6 (Ce6) modified silicon nanoparticles (SiNPs)	ABC transporter pathway	Fluorescence imaging and photodynamic killing of Gram-negative and Gram-positive bacteria
Nat. Commun. **13**, 1255 (2022)	GP and Ce6 modified gold nanoparticles (AuNPs)	ABC transporter pathway	Photoacoustic imaging and photodynamic and photothermal killing of Gram-negative and Gram-positive bacteria
Nat. Commun. **13**, 5127 (2022)	GP and indocyanine green (ICG) modified SiNPs	ABC transporter pathway	A bacteria-based drug delivery system for glioblastoma photothermal immunotherapy
Angew. Chem. Int. Ed. **61**, e202208422 (2022)	GP and ICG modified SiNPs, AuNPs or carbon dots (CDs)	ABC transporter pathway	A bacteria-based drug delivery system for photothermal programmable destruction of deep tumor tissues
This work	GP, luciferase, Cy5 and ICG-modified SiNPs, GP and D-luciferin-modified SiNPs	ABC transporter pathway	Bioluminescence imaging and photothermal killing of Gram-negative and Gram-positive bacteria

Supplementary Table 2. Blood biochemical analysis of health mice treated with PBS, GP-Si-Luc or GP-Si-BPs for 24 h.

Analysis index	PBS		GP-Si-Luc		GP-Si-BPs	
	Mean	Standard deviation	Mean	Standard deviation	Mean	Standard deviation
Albumin and globulin ratio	2.15	0.11	2.18	0.13	1.81	0.12
Alanine aminotransferase (U/L)	14.04	0.84	18.59	1.19	13.26	3.40
Aspartate aminotransferase (U/L)	45.53	7.49	46.43	10.08	47.19	7.08
Alkaline phosphatase (U/L)	46.88	13.81	46.20	4.35	52.66	3.52
Urea (mmol/L)	10.64	0.10	10.51	0.26	10.61	0.33
Creatinine (μmol/L)	6.47	0.63	9.06	1.78	8.04	0.67
Total protein (mmol/L)	18.54	0.56	17.70	0.45	18.70	0.70
Albumin (g/L)	12.66	0.34	12.12	0.09	12.02	0.17
Globulin (g/L)	5.89	0.33	5.58	0.38	6.68	0.54

Supplementary Table 3. Blood routine analysis of mice injected with PBS, GP-Si-Luc or GP-Si-BPs for 24 h.

Analysis index	PBS		GP-Si-Luc		GP-Si-BPs	
	Mean	Standard deviation	Mean	Standard deviation	Mean	Standard deviation
White blood cell (1.0×10^3 cells/μL)	0.62	0.27	0.89	0.12	0.63	0.20
Red blood cell (1.0×10^6 cells/μL)	1.96	1.56	1.96	1.24	8.03	2.12
Hemoglobin (g/dL)	2.77	0.49	2.77	0.32	3.13	0.51
Hematocrit (%)	11.53	2.27	11.30	1.21	13.17	2.61
Mean corpuscular volume (fL)	59.10	0.62	57.70	1.42	59.37	1.19
Mean corpuscular hemoglobin (pg)	14.03	0.38	14.37	0.32	14.17	0.55
Mean corpuscular hemoglobin concentration (g/dL)	23.80	0.82	24.83	0.58	23.83	0.55
Mean corpuscular hemoglobin concentration (g/dL)	22.53	0.15	23.03	0.40	22.37	0.35
Cholesterol (pg)	13.27	0.15	13.27	0.25	13.23	0.06
Red cell volume distribution width (%)	12.33	0.12	12.03	0.25	12.30	0.35

Hemoglobin content distribution width (g/dL)	1.71	0.02	1.68	0.04	1.68	0.06
Platelet (1.0×10^3 cells/μL)	109.33	13.05	100.00	8.72	108.33	4.51
Mean platelet volume (fL)	10.13	0.72	9.13	0.42	10.03	0.32

Supplementary Notes 1

Calculation of FRET efficiency and BRET ratio.

FRET efficiencies between Cy5 and ICG in GP-Si-BPs were calculated according to the following equation:

$$E = 1 - F'_D/F_D \qquad (1)$$

where F'_D and F_D were the donor fluorescence intensities with and without an acceptor, respectively. The optimal FRET efficiency between Cy5 and ICG was calculated to be 32% (**Fig. 3c**).

The Förster distances (R_0) of these two steps were also calculated to be 1.03 nm according to the following equations:

$$J(\lambda) = [\int_0^{\infty} F_D(\lambda)\varepsilon_A(\lambda)\lambda^4 d\lambda]/[\int_0^{\infty} F_D(\lambda)d\lambda] \qquad (2)$$

$$R_0 = 0.0211[\kappa^2 n^{-4}\Phi_D J(\lambda)]^{1/6} \qquad (3)$$

where $F_D(\lambda)$ was the area-normalized emission spectrum of donor, $\varepsilon_A(\lambda)$ was the molar absorption spectrum of the acceptor in $M^{-1}cm^{-1}$, λ was the wavelength in nm, κ^2 was orientation factor ($\kappa^2 = 2/3$ due to dynamic averaging donor-acceptor systems), Φ_D was quantum yield of the donor, and n = 1.35 was the refractive index of the surrounding medium.

Supplementary Notes 2

Confirmation of lamB knockout by Sanger sequencing

AAAGCCGTGATGTCCAGGTTGGAGCCAATATGTCGCTGGGTATTCGCCCGG
AACATCTACTGCCGAGTGATATCGCTGACGTCATCCTTGAGGGTGAAGTTC
AGGTCGTCGAGCAACTCGGCAACGAAACTCAAATCCATATCCAGATCCCTT
CCATTCGTCAAAACCTGGTGTACCGCCAGAACGACGTGGTGTTGGTAGAA
GAAGGTGCCACATTCGCTATCGGCCTGCCGCCAGAGCGTTGCCATCTGTTC
CGTGAGGATGGCACTGCATGTCGTCGACTGCATAAGGAGCCGGGCGTTTAA
GCACCCCACAAAACACACAAAGCCTGTCACAGGTGATGTGAAAAAAGAA
AAGCAATGACTCAGGAGATAGATAGCAAAACCTGGGCCGGATAAGGCGTT
TACGCCGCATTCGGCAACCAACGCCTGATGCGACGCTTGCGCGTCTTATCA
GGCCTACAACGGCTGTCAAATGTAGGCCGGATAAGGCGTTTACGCCGCATC
CGGCATAAAAACAGGTTGTCATTATCTGAAAGGGGCGAAAGCCCCTCTGAT
TATCGGGTTTAGCGCGCTATTGCCTGGCTACCGCTGAGCTCCAGATTTTGAG
GTGAAAACAATGAAAATGAATAAAAGTCTCATCGTCCTCTGTTTATCAGCA
GGGTTACTGGCAAGCGCGCCTGGAATTAGCCTTGCCGATGTTAACTACGTA
CCGCAAAACACCAGCGACGCGCCAGCCATTCCATCTGCTGCGCTGCAACA
ACTCACCTGGACACCGGTCGATCAATCT

Supplementary Notes 3

Confirmation of malE knockout by Sanger sequencing

GCTGTACGCTCGCCATGCCCTTCTCCCTTTGTAACAACCTGTCATCGACAGC
AACATTCATGATGGGCTGACTATGCGTCATCAGGAGATGGCTTAAATCCTCC
ACCCCCTGGCTTTTTTATGGGGGAGGAGGCGGGAGGATGAGAACACGGCT
TCTGTGAACTAAACCGAGGTCATGTAAGGAATTTCGTGATGTTGCTTGCAA
AAATCGTGGCGATTTTATGTGCGCATCTCCACATTACCGCCAATTCTGTAAC
AGAGATCACACAAAGCGACGGTGGGGCGTAGGGGCAAGGAGGATGGAAA
GAGGTTGCCGTATAAAGAAACTAGAGTCCGTTTAGGTGTTTTCACGAGCAC
TTCACCAACAAGGACCATAGATTTGCTGTGAAATGCCGGATGCGGCGTGAA
CGCCTTGTCCGGCCTACAAAACCGAAACGTATGTAGGCCTGATAAGACGCG
TCAGCGTCGCATCAGGCAGTTGTTGTCGGATAAGGCGTGAAAGCCTTATCC
GTCCTGGAATGAGGAAGAACCCCATGGATGTCATTAAAAAGAAACATTGGT
GGCAAAGCGACGCGCTGAAATGGTCAGTGCTAGGTCTGCTCGGCCTGCTG
GTGGGTTACCTTGTTGTTTTAATGTACGCACAAGGGGAATACCTGTTCGCCA
TTACCACGCTGATATTGAGTTCAGCGGGGCTGTATATTTTCGCCAATCGTAA
AGCCTACGCCTGGCGCTATGTTTACCCGGGAATGGCTGGAATGGGATTATTC
GTCCTCTTCCCTCTGGTCTGCACCATCGCCATTGCCTTCACCA
