# Supplementary material for: In vivo bioluminescence imaging of natural bacteria within deep tissues via ATP-binding cassette sugar transporter
Source: Nat Commun. 2023 Apr 22;14:2331. doi: 10.1038/s41467-023-37827-9 (PMC10122673; doi:10.1038/s41467-023-37827-9)
Supplement: Supplementary file 3 — Reporting Summary [file 41467_2023_37827_MOESM3_ESM.pdf]

nature portfolio

Corresponding author(s): Jiaxu Hong, Houyu Wang, Yao He

Last updated by author(s): Apr 2, 2023

Reporting Summary

Nature Portfolio wishes to improve the reproducibility of the work that we publish. This form provides structure for consistency and transparency in reporting. For further information on Nature Portfolio policies, see our Editorial Policies and the Editorial Policy Checklist.

Statistics

For all statistical analyses, confirm that the following items are present in the figure legend, table legend, main text, or Methods section.

n/a	Confirmed	
☐	☒	The exact sample size (*n*) for each experimental group/condition, given as a discrete number and unit of measurement
☐	☒	A statement on whether measurements were taken from distinct samples or whether the same sample was measured repeatedly
☐	☒	The statistical test(s) used AND whether they are one- or two-sided<br>*Only common tests should be described solely by name; describe more complex techniques in the Methods section.*
☒	☐	A description of all covariates tested
☒	☐	A description of any assumptions or corrections, such as tests of normality and adjustment for multiple comparisons
☐	☒	A full description of the statistical parameters including central tendency (e.g. means) or other basic estimates (e.g. regression coefficient) AND variation (e.g. standard deviation) or associated estimates of uncertainty (e.g. confidence intervals)
☐	☒	For null hypothesis testing, the test statistic (e.g. *F*, *t*, *r*) with confidence intervals, effect sizes, degrees of freedom and *P* value noted<br>*Give P values as exact values whenever suitable.*
☒	☐	For Bayesian analysis, information on the choice of priors and Markov chain Monte Carlo settings
☒	☐	For hierarchical and complex designs, identification of the appropriate level for tests and full reporting of outcomes
☒	☐	Estimates of effect sizes (e.g. Cohen's *d*, Pearson's *r*), indicating how they were calculated

Our web collection on statistics for biologists contains articles on many of the points above.

Software and code

Policy information about availability of computer code

Data collection	Leica Application Suite Advanced Fluorescence Lite (LAS AF Lite) 2.6.0<br>Malvern ZETASIZER NANO<br>C6 Plus Flow Cytometry<br>IVIS Lumina III In Vivo Imaging System
Data analysis	Softwares used in analysis include Origin 2018, Graphpad Prism 9.0

For manuscripts utilizing custom algorithms or software that are central to the research but not yet described in published literature, software must be made available to editors and reviewers. We strongly encourage code deposition in a community repository (e.g. GitHub). See the Nature Portfolio guidelines for submitting code & software for further information.

Data

Policy information about availability of data

All manuscripts must include a data availability statement. This statement should provide the following information, where applicable:

- Accession codes, unique identifiers, or web links for publicly available datasets
- A description of any restrictions on data availability
- For clinical datasets or third party data, please ensure that the statement adheres to our policy

The data that support the findings of this study are available within the paper and its supplementary information. Source data underlying Fig. 2c, Fig. 3a, 3c, 3d, 3e,

3f, 3h, Fig. 5a, 5b, 5c, 5d, Fig. 6a, 6c, 6d, Fig. 7b, 7e, Supplementary Fig. 2a, 2b, 2c, 2d, 2e, Supplementary Fig. 3a, 3b, 3c, Supplementary Fig. 4, Supplementary Fig. 5a, 5b, 5c, 5d, Supplementary Fig. 7a, 7b, Supplementary Fig. 8, Supplementary Fig. 9, Supplementary Fig. 16e, Supplementary Fig. 20e, Supplementary Fig. 22e, Supplementary Fig. 23d and Supplementary Fig. 25b are provided as a Source Data file. Any other data are available from the authors upon reasonable request.

Human research participants

Policy information about studies involving human research participants and Sex and Gender in Research.

Reporting on sex and gender	The authors state that gender was chosen at random and has nothing to do with the results of this experiment
Population characteristics	Human-derived MDR E. coli and MRSA were isolated from patients with keratitis. Human blood samples were provided by a healthy volunteer following written informed consent. Ten patients with bacterial endophthalmitis who were undergoing vitreous surgery, uncontaminated non-diluted vitreous fluid samples (0.1 mL) were collected in a syringe with a 30 G needle during diagnostic pars plana vitrectomy (PPV). Immediately after collection, the sample was transferred into a pre-sterilized microfuge tube and used for imaging. These 10 patients included 6 men (aged 32, 46, 37, 43, 39, and 36 years) and 4 women (aged 60, 63, 30, and 28 years).
Recruitment	Blood was donated from the primary researchers and did not involve recruitment or enrollment of human subjects. The authors also state that informed consent was obtained from all participants for any experiments with human samples without any identifying information. The Eye, Ear, Nose and Throat Hospital, Fudan University, is committed to the protection and safety of human samples involved in the research.
Ethics oversight	The study protocols using human blood samples were approved by the ethics committee of Soochow University. The authors state that all human blood experiments were performed in strict accordance with the relevant laws and institutional guidelines. The clinical samples were supplied by the Eye Bank of the Eye, Ear, Nose and Throat Hospital, Fudan University, under the approval of the hospital ethics committee (EENTIRB-2017-06-07-01). All experiments were conducted according to the Declaration of Helsinki and in compliance with Chinese law.

Note that full information on the approval of the study protocol must also be provided in the manuscript.

Field-specific reporting

Please select the one below that is the best fit for your research. If you are not sure, read the appropriate sections before making your selection.

☒ Life sciences ☐ Behavioural & social sciences ☐ Ecological, evolutionary & environmental sciences

For a reference copy of the document with all sections, see nature.com/documents/nr-reporting-summary-flat.pdf

Life sciences study design

All studies must disclose on these points even when the disclosure is negative.

Sample size	Group sizes for experiments were chosen on the basis of prior experience and literature precedence, so that sufficient numbers were used to ensure reproducibility and determine standard deviations. The number of animals was at least 3. For each sample, two technical replicates were carried out. If there was 20% or greater variation between technical replicates, an additional two technical replicates were carried out. . Sample sizes employed in this study were referenced previously published studies (Nature Communications 2022, 13: 1255).
Data exclusions	No data were excluded from the analyses.
Replication	All experiments were carried out with at least 3 replicate samples for each experimental group, and we confirmed all attempts at replication were successful.
Randomization	Samples were allocated into experimental groups at random.
Blinding	All the data collection and analysis were from blinded with randomized samples.

Reporting for specific materials, systems and methods

We require information from authors about some types of materials, experimental systems and methods used in many studies. Here, indicate whether each material, system or method listed is relevant to your study. If you are not sure if a list item applies to your research, read the appropriate section before selecting a response.

Materials & experimental systems

n/a	Involved in the study
☒	☐ Antibodies
☐	☒ Eukaryotic cell lines
☒	☐ Palaeontology and archaeology
☐	☒ Animals and other organisms
☒	☐ Clinical data
☒	☐ Dual use research of concern

Methods

n/a	Involved in the study
☒	☐ ChIP-seq
☐	☒ Flow cytometry
☒	☐ MRI-based neuroimaging

Eukaryotic cell lines

Policy information about cell lines and Sex and Gender in Research

Cell line source(s)	HEK-293T, HeLa, MCF-7 and mREC cells were sourced form Shanghai Zhong Qiao Xin Zhou Biotechnology Co., Ltd (China)
Authentication	Cell lines were used from the source without authentication.
Mycoplasma contamination	Cells were tested monthly and found to be negative for mycoplasma contamination.
Commonly misidentified lines (See ICLAC register)	No commonly misidentified cell lines were used.

Animals and other research organisms

Policy information about studies involving animals; ARRIVE guidelines recommended for reporting animal research, and Sex and Gender in Research

Laboratory animals	For small-animal studies, nude mice (SPF grade, 6-8 weeks old) were used.
Wild animals	No wild animals were involved in the study .
Reporting on sex	All mice involved in the study were female.
Field-collected samples	No field-collected samples were used in the study.
Ethics oversight	All animal experimental procedures were performed according to the Guideline for Animal Experimentation with the approval of the animal care committee of Soochow University.

Note that full information on the approval of the study protocol must also be provided in the manuscript.

Flow Cytometry

Plots

Confirm that:

☒ The axis labels state the marker and fluorochrome used (e.g. CD4-FITC).

☒ The axis scales are clearly visible. Include numbers along axes only for bottom left plot of group (a 'group' is an analysis of identical markers).

☒ All plots are contour plots with outliers or pseudocolor plots.

☒ A numerical value for number of cells or percentage (with statistics) is provided.

Methodology

Sample preparation	MRSA and MDR E. coli was co-incubated with probes of GP-Si-BPs with GP concentration (0, 0.14, 0.28, 0.56, 1.12 mM) at 37 oC for 0.5 h, 1.5 h, 2.5 h and 3.5 h, respectively. S. aureus, Salmonella typhimurium (STm) or Bacteria mutants of ΔlamB and ΔmalE was co-incubated with 0.06 mM GP-Si-BPs and 0.06 mM GP-Si-Luc for 2.5 h. Then, the samples were collected and washed with PBS with centrifugation (3381 x g, 5 min). The samples were resuspended in PBS in a flow tube. The ratios of bacterial cells that uptake probes in the total bacterial cells were detected by flow cytometry.
Instrument	BD C6 Plus Flow Cytometry
Software	FlowJo_V10
Cell population abundance	20000 bacterial cells in gate

Gating strategy

Bacterial cells were gated by FSC and SSC plots to obtain a live population.

☐ Tick this box to confirm that a figure exemplifying the gating strategy is provided in the Supplementary Information.
